# Pocket hematoma after pacemaker or defibrillator surgery: Direct oral anticoagulants versus vitamin K antagonists

**DOI:** 10.1016/j.ijcha.2022.101005

**Published:** 2022-03-16

**Authors:** John de Heide, Marisa van der Graaf, Marijn J. Holl, Rohit E. Bhagwandien, Dominic A.M.J. Theuns, André de Wit, Felix Zijlstra, Tamas Szili-Torok, Mattie J. Lenzen, Sing-Chien Yap

**Affiliations:** Department of Cardiology, Erasmus MC, University Medical Center Rotterdam, Rotterdam, the Netherlands. All authors take responsibility for all aspects of the reliability and freedom from bias of the data presented and their discussed interpretation

**Keywords:** Atrial fibrillation, Bleeding, Direct oral anticoagulant, Implantable cardioverter defibrillator, non-vitamin K antagonist, Pacemaker

## Abstract

**Background:**

Direct oral anticoagulants (DOACs) are the preferred choice of oral anticoagulation in patients with atrial fibrillation (AF). Randomized trials have demonstrated the efficacy and safety of DOAC in patients undergoing a cardiac implantable electronic device procedure (CIED); however, there is limited real-world data.

**Objective:**

To evaluate the outcome of patients undergoing an elective CIED procedure in a tertiary referral center with an interrupted DOAC or continued vitamin K antagonist (VKA) regimen.

**Methods:**

This was a retrospective single-center study of consecutive patients with AF undergoing an elective CIED procedure between January 2016 and June 2019. The primary endpoint was a clinically significant pocket hematoma < 30 days after surgery. The secondary endpoint was any systemic thromboembolic complication < 30 days after surgery.

**Results:**

Of a total of 1,033 elective CIED procedures, 283 procedures were performed in patients with AF using oral anticoagulation. One-third of the procedures were performed under DOAC (N = 81, 29%) and the remainder under VKA (N = 202, 71%). The DOAC group was younger, had less chronic renal disease, more paroxysmal AF and a lower HAS-BLED score. The VKA group more often underwent a generator change only in comparison to the DOAC group. Clinically significant pocket hematoma occurred in 5 patients (2.5%) in the VKA group and did not occur in the DOAC group (*P* = 0.33). There were no thromboembolic events reported.

**Conclusion:**

In patients with AF undergoing an elective CIED procedure, the risk of a pocket hematoma and a systemic thromboembolic event is comparably low when using either continued VKA or interrupted DOAC.

## 1 Introduction

In patients with atrial fibrillation (AF) direct oral anticoagulants (DOACs) are currently the preferred choice of oral anticoagulation for long-term stroke prevention.[1], [2] A cardiac implantable electronic device (CIED) procedure is generally considered a procedure with a low bleeding risk.[2] However, device-pocket hematoma is a common complication with an incidence ranging from 0.2% up to 16%, depending on definition and antithrombotic regimen.[3], [4], [5], [6], [7] A pocket hematoma is associated with local discomfort, increased risk of infection, prolongation of hospitalization and may require surgical intervention in some cases.[8], [9], [10], [11]

Previous studies have shown that periprocedural oral anticoagulation is associated with a higher likelihood for pocket hematoma.[5], [12], [13] The current guidelines recommend continuation of vitamin K antagonists (VKAs) during CIED procedures as bridging therapy with heparin is associated with a five-fold higher risk of bleeding compared with continued VKA.[2], [4], [7] With regard to periprocedural DOAC use, the BRUISE CONTROL-2 trial, published in 2018, demonstrated that continued and interrupted DOAC had a similar low incidence of clinically significant pocket hematoma.[3] However, a *meta*-analysis in 2020 demonstrated a numerically higher incidence of bleeding complications in patients who continued DOAC.[14] Furthermore, a large European survey demonstrated that in the majority of patients (89%) an interrupted DOAC strategy was used.[15] The ESC guidelines and a EHRA expert consensus statement did not suggest a preference for either continued or interrupted DOAC during CIED surgery.[6], [7] Currently, there is little real-world data comparing the safety and efficacy of continued VKA versus interrupted DOAC in patients undergoing CIED surgery. The aim of the present study is to evaluate the incidence of clinically significant device pocket hematoma between both periprocedural anticoagulation regimens in patients with AF undergoing an elective CIED procedure in an academic center.

## 2 Methods

### 2.1 Study cohort

We retrospectively evaluated all consecutive adult patients who underwent an elective pacemaker or defibrillator surgery between January 2016 and June 2019. This population did not include patients with a recent (<3 months) transvenous lead extraction, patients who received a device during unplanned hospitalization, and patients who received a leadless pacemaker. The only inclusion criterion was a history of AF. Exclusion criteria were the use of concomitant antiplatelet therapy (i.e., aspirin, clopidogrel, ticagrelor or prasugrel) and any other regimen than continued VKA or interrupted DOAC. Thus, patients with bridging therapy, interrupted VKA or no oral anticoagulation use were excluded. No patient in our center continued DOAC during an elective CIED procedure. Data were collected from the electronic medical records.

### 2.2 Anticoagulation regimen and discharge

Patients using DOAC discontinued their drug 24–48 h before surgery depending on their renal function. All DOACs were restarted 24 h after end of surgery, unless stated otherwise by the operator. In patients using acenocoumarol or fenprocoumon, the target international normalized ratio (INR) was 2.0 to 2.5 in the morning of the procedure. Patients with continued VKA usually attained to their regular dosing schedule.

Patients undergoing a device implantation were discharged the day after the procedure. At the day of discharge, these patients underwent a physical examination of their device pocket, had a device interrogation and a chest X-ray. Patients undergoing a generator replacement only were discharged on the same day of the procedure after clinically significant pocket hematoma had been ruled out.

### 2.3 Study endpoints

The primary endpoint was clinically significant device pocket hematoma < 30 days after surgery. A clinically significant hematoma was defined as a hematoma resulting in either re-operation, prolongation of hospitalization (>24 h after index surgery) or interruption of oral anticoagulation. This definition of clinically significant hematoma is in accordance with the landmark BRUISE CONTROL trials.[4], [16] The secondary endpoint was any systemic thromboembolic complication (i.e., transient ischemic attack, stroke) < 30 days after surgery.

### 2.4 Statistical analysis

Continuous parameters were tested for normality before analysis and are expressed as mean ± standard deviation (SD) or median (interquartile range), as appropriate. Categorical data are presented as frequencies and percentages. Comparisons between groups were performed with an independent Student *t*-test, chi-square tests, Fisher exact test, or a Mann-Whitney *U* test, as appropriate. All analyses were two-tailed; a p-value < 0.05 was considered statistically significant. Statistical analyses were performed using SPSS software (SPSS, version 25; IBM, Chicago, Illinois).

### 2.5 Ethics

The Medical Ethics Committee reviewed the study (MEC-2020–0299), and this study was not subjected to the Dutch Medical Research Involving Human Subjects Act. The study was carried out according to the ethical principles for medical research involving human subjects established by Declaration of Helsinki, protecting the privacy of all the participants and the confidentiality of their personal information.

## 3 Results

### 3.1 Study population

**Fig. 1:**
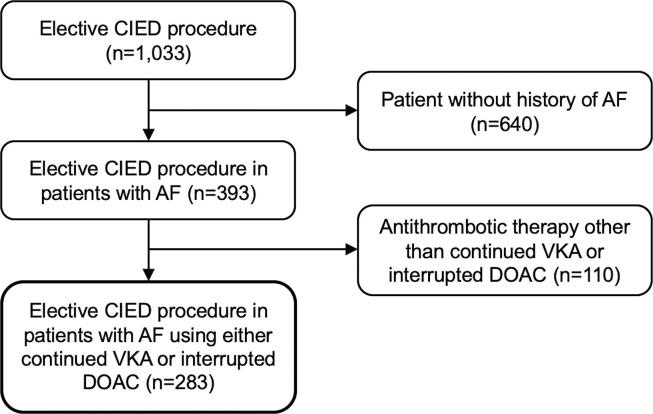
Flow chart study population. Abbreviations: AF = atrial fibrillation, CIED = cardiac implantable electronic device; DOAC = direct oral anticoagulant; VKA = vitamin K antagonist.

**Fig. 2:**
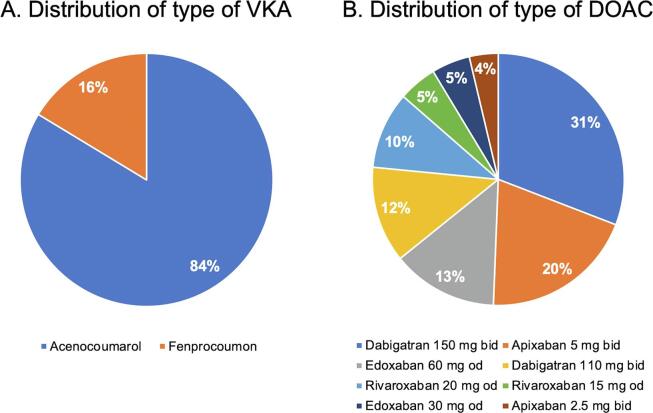
Distribution of type and dose of periprocedural oral anticoagulation. Abbreviations: DOAC = direct oral anticoagulant; VKA = vitamin K antagonist.

**Fig. 3:**
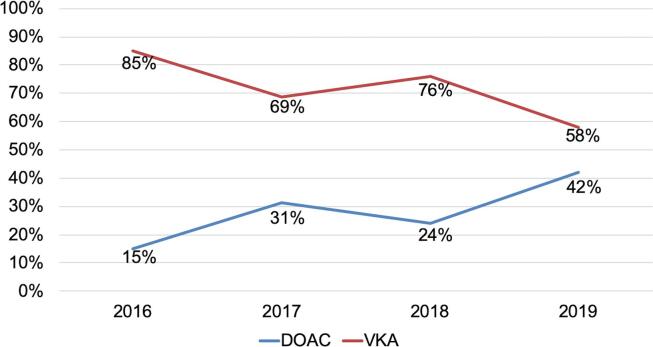
Temporal trend in the type of periprocedural oral anticoagulation. Abbreviations: DOAC = direct oral anticoagulant; VKA = vitamin K antagonist.

A total of 1,033 elective CIED procedures were performed during the study period. After exclusion of patients who did not fulfil the criteria, the final study population consisted of 283 patients (Fig. 1). The VKA group comprised 202 patients (71%) and the DOAC group comprised 81 patients (29%). In the VKA group, most patients used acenocoumarol (Fig. 2A). In the DOAC group, most patients used dabigatran (43%) or apixaban (24%) (Fig. 2B). Patients who used a lower dose of DOAC had a lower mean eGFR in comparison to those with a normal dose of DOAC (50 ± 23 mL/min vs 74 ± 18 mL/min, p= < 0.001). The use of DOAC in the study population increased over the years, increasing from 15% in 2016 to 42% in 2019 (Fig. 3).

Baseline patient characteristics are depicted in Table 1. In comparison to the VKA group, patients using DOACs were younger, had a lower median HAS-BLED score, were more likely to have paroxysmal AF and to use class I antiarrhythmic drugs, but less likely to have chronic renal disease and to use digoxin and diuretics. Patients with mechanical heart valves were only present in the VKA group. In the VKA group, the median INR at the day of surgery was 2.1 (IQR 1.8–2.4). In the DOAC group, the rhythm at the day of the procedure was sinus rhythm (57%), AF (38%), atrial flutter (3%) and atrioventricular sequential pacing (3%).

**Table 1:** Baseline characteristics.

**Characteristic**	**VKA group (n = 202)**	**DOAC group (n = 81)**	**P-value**
Age in years	71 (63–77)	68 (62–73)	0.04
Male sex	144 (71.3%)	52 (64.2%)	0.24
Body mass index	26.0 (23.7–30.1)	27.3 (23.9–30.0)	0.27
Medical history			
- Chronic heart failure	134 (66.3%)	49 (60.5%)	0.35
- Hypertension	79 (39.1%)	34 (42.0%)	0.66
- Diabetes mellitus	32 (15.8%)	13 (16.0%)	0.97
- Stroke	22 (10.9%)	4 (49%)	0.12
- Transient ischemic attack	26 (12.9%)	5 (6.2%)	0.10
- Coronary artery disease	69 (34.2%)	25 (30.9%)	0.59
- Peripheral artery disease	14 (6.9%)	7 (8.6%)	0.62
- Chronic renal disease*	105 (52.0%)	29 (35.8%)	0.01
- eGFR (mL/min)	56 ± 22	68 ± 22	<0.001
- Dilated cardiomyopathy	61 (30.2%)	28 (34.6%)	0.47
- Ischemic cardiomyopathy	53 (26.2%)	16 (19.8%)	0.25
- COPD	42 (20.8%)	13 (16.0%)	0.36
- Mechanical heart valve	23 (11.4%)	–	0.002
- History of bleeding	15 (7.4%)	4 (4.9%)	0.45
Type of AF:			0.034
- Paroxysmal AF	98 (48.5)	53 (65.4)	
- Persistent AF	30 (14.9)	9 (11.1)	
- Permanent AF	74 (36.6)	19 (23.5)	
CHA_2_DS_2_-VASc score	3 (2–5)	3 (2–4)	0.07
HAS-BLED score	2 (1–3)	1 (1–2)	<0.001
Cardiac medication:			
- ACEI	88 (43.6%)	26 (32.1%)	0.08
- ARB	56 (27.7%)	18 (22.2%)	0.34
- Aldosterone inhibitor	81 (40.1%)	23 (28.4%)	0.07
- Digoxin	56 (27.7%)	13 (16.0%)	0.04
- Class I AAD	6 (3.0%)	8 (9.9%)	0.03
- Beta-blocker	141 (69.8%)	53 (65.4%)	0.47
- Amiodarone	48 (23.8%)	18 (22.2%)	0.78
- Sotalol	15 (7.4%)	4 (4.9%)	0.45
- Calcium antagonist	26 (12.9%)	8 (9.9%)	0.48
- Diuretics	138 (68.3%)	35 (43.2%)	<0.001
- Statin	111 (55.0%)	36 (44.4%)	0.11

Data are presented as n (%), median (25th, 75th percentile) or mean ± standard deviation. * eGFR < 60 mL/min. Abbreviations: AAD = antiarrhythmic drug; ACEI = angiotensin-converting-enzyme inhibitor; AF = atrial fibrillation; ARB = angiotensin receptor blocker; COPD = chronic obstructive pulmonary disease; DOAC = direct oral anticoagulants; eGFR = estimated glomerular filtration rate; OAC = oral anticoagulant; VKA = vitamin-K antagonist.

Besides differences in patient characteristics, there were also differences in surgical characteristics (Table 2). The DOAC group more often underwent a de novo dual chamber device implantation, while the VKA group more often had a pulse generator replacement procedure only. The median procedure duration was longer in the DOAC group in comparison to the VKA group.

**Table 2:** Operative details.

**Characteristic**	**VKA group(n = 202)**	**DOAC group (n = 81)**	**P-value**
New implant of a pacemaker			
- Single	6 (3.0)	4 (4.9)	0.42
- Dual	16 (7.9)	19 (23.5)	<0.001
- Cardiac resynchronization	9 (4.5)	4 (4.9)	0.86
New implant of an ICD			
- Single	9 (4.5)	6 (7.4)	0.32
- Dual	3 (1.5)	5 (6.2)	0.03
- Cardiac resynchronization	11 (5.4)	3 (3.7)	0.54
- Subcutaneous ICD	4 (2.0)	4 (4.9)	0.17
Device replacement or revision			
- Pulse generator change only	115 (56.9)	21 (25.9)	<0.001
- Pulse generator change with additional	25 (12.4)	12 (14.8)	0.58
- Other	4 (2.0)	3 (3.7)	0.40
Subpectoral pocket	15 (7.4)	5 (6.2)	0.71
INR at day of procedure	2.1 (1.8–2.4)	–	
Duration of procedure (min)	50 (32–75)	69 (45–91)	0.003

Data are presented as n (%) or as median (25th, 75th percentile). Abbreviations: ICD = implantable cardioverter-defibrillator; DOAC = direct oral anticoagulant; INR = international normalized ratio, VKA = vitamin K antagonist.

### 3.2 Study endpoints

**Fig. 4:**
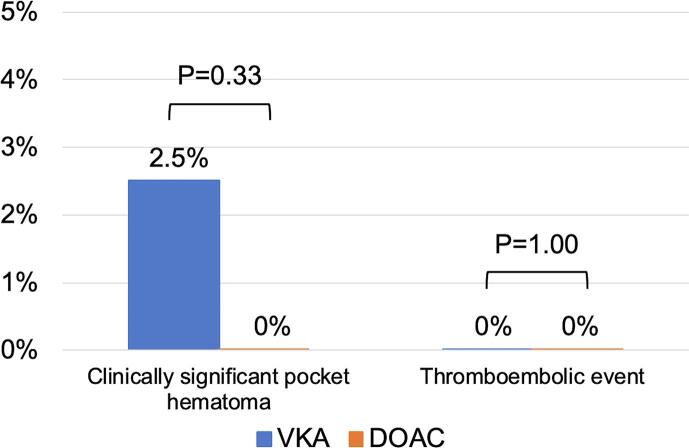
Primary and secondary outcomes. Abbreviations: DOAC = direct oral anticoagulant; VKA = vitamin K antagonist.

The primary endpoint occurred only in the VKA group. Although, there was a numerically higher incidence of clinically significant pocket hematoma in the VKA group (2.5%, 95% confidence interval [CI] 0.8%–5.7%) in comparison to the DOAC group (0%, 95% CI 0%–4.5%), this was not statistically different (*P* = 0.33) (Fig. 4). Of the 5 patients with clinically significant pocket hematoma, 4 patients (80%) had a device replacement or revision as the index procedure, 3 patients (60%) had an impaired renal function (eGFR < 60 mL/min) at baseline and 3 of 5 patients (60%) were > 70 years of age at the time of surgery (Table 3). Only 1 patient with a clinically significant pocket hematoma required a reoperation. Regarding the secondary endpoint, no systemic thrombotic event occurred (Fig. 4).

**Table 3:** Detailed overview of clinically significant hematoma.

**Case no.**	**Type VKA**	**Sex**	**Age**	**Cardiomyopathy**	**eGFR (mL/min)**	**HAS-BLED**	**Type of procedure**	**Intervention**
1	Acenocoumarol	M	64	Ischemic	67	2	Generator change and ICD lead implantation	Prolongation of hospital stay & interruption of VKA
2	Acenocoumarol	M	66	Non-ischemic	5	3	CRT-D implantation	Interruption of VKA
3	Acenocoumarol	F	72	none	60	2	RV pacing lead change	Prolongation of hospital stay
4	Acenocoumarol	M	81	Ischemic	56	3	ICD generator change	Prolongation of hospital stay
5	Acenocoumarol	M	88	none	34	2	ICD generator change	Interruption of VKA & reoperation

Data are presented as n (%). Abbreviations: CRT-D = cardiac resynchronization therapy defibrillator; eGFR = estimated glomerular filtration rate; F = female; ICD = implantable cardioverter-defibrillator; M = male; VKA = vitamin K antagonist.

## 4 Discussion

The present study demonstrates that continued VKA and interrupted DOAC were associated with a comparable low risk of clinically significant pocket hematoma in patients with AF undergoing CIED surgery in a tertiary referral center. Furthermore, no systemic thromboembolic events were observed in both groups in the first month after surgery.

### 4.1 Pocket hematoma and periprocedural anticoagulation

Pocket hematoma is one of the most common complications following CIED surgery.[6] A pocket hematoma is not always benign and can be associated with prolongation of hospitalization, an increased risk of reoperation, and serious device-related infection.[8], [10], [11], [17] Therefore, prevention of pocket hematoma is important and this requires meticulous attention to modifiable risk factors, good operative skills and proper patient preparation. Risk factors for device pocket hematoma includes older age, renal failure, congestive heart failure, low operator experience, concomitant antiplatelet therapy, device replacement, lead revision, and heparin bridging.[4], [15], [17], [18], [19], [20], [21], [22], [23] In patients using VKA, continued VKA is preferred over heparin bridging as the last is associated with a higher risk of pocket hematoma and prolonged hospital stay.[4], [6], [19] Currently, most centers prefer either a continued VKA regimen or interrupt VKA without heparin bridging in case of a low CHADS-VASc score (<3) in patients with AF.[15]

With regard to periprocedural DOAC, the 2021 ESC guidelines on Cardiac Pacing and Cardiac Resynchronization Therapy and a 2021 EHRA expert consensus statement have no specific preference for either continued or interrupted DOAC in patients undergoing CIED surgery.[6], [7] The BRUISE CONTROL-2 trial demonstrated a similar low risk for clinically significant pocket hematoma in patients using either continued or interrupted DOAC (2.1% in both groups).[3] Several single-center studies have demonstrated a similar low risk of clinically significant pocket hematoma when using continued DOAC,[24], [25] however, a recent *meta*-analysis demonstrated a numerically higher incidence of bleeding complications in patients who continued DOAC.[14] Furthermore, many centers still prefer a interrupted DOAC regimen.[15]

Therefore, it is interesting to know in a real-world population how an interrupted DOAC regimen would compare to the widely accepted continued VKA regimen regarding the incidence of pocket hematoma. It should be noted that we excluded patients who used concomitant antiplatelet therapy to prevent bias, as it is well-established that concomitant antiplatelet therapy in anticoagulated patients is associated with a two-fold higher risk of clinically significant pocket hematoma.[23] We observed a low incidence of clinically significant pocket hematoma; this was 2.5% in patients using continued VKA and 0% in patients with interrupted DOAC. Our results are in line with both BRUISE CONTROL trials, which showed an incidence of 3.5% and 2.1% in the continued VKA arm and interrupted DOAC arm, respectively.[3], [4] Using patient level data from both BRUISE CONTROL trials, Essebag *et al*. also showed no difference in clinically significant pocket hematoma between DOAC use (either continued or interrupted) and continued VKA after adjusting for concomitant antiplatelet use (odds ratio 0.86, 95% CI 0.38–1.96, *P* = 0.72).[23]

### 4.2 Trend in DOAC use

In the Netherlands, there was initially a conservative policy with regard to DOAC use, mainly due to concerns about the lack of an antidote, patient adherence, lack of monitoring and increased health care costs.[26] Therefore, there was a slower uptake of DOAC use in the Netherlands in comparison to other Western European countries.[27] Since 2016 there has been a steady increase in the use of DOAC in the Netherlands. This is reflected by the steady increase in the relative proportion of patients with periprocedural DOAC in our study population, from 15% in 2016 to 42% in 2019. This also explains why patients in the DOAC group were more likely to undergo a de novo implantation and less likely to undergo a device replacement in comparison to the VKA group. Because a device replacement is associated with a higher likelihood of pocket hematoma,[21] this may result in bias towards a more favourable outcome for the DOAC group in comparison to the VKA group in the present study.

It is expected that in the future the majority of patients will undergo CIED surgery with periprocedural DOACs as these are the preferred agents for stroke prevention in patients with AF.[1] Also, the potential treatment of device-detected AF with DOAC, depending on the outcome of NOAH-AFNET 6 and ARTESiA,[28], [29] will result in more CIED patients being treated with a DOAC. Our real-world data is reassuring that an interrupted DOAC regimen is associated with a low risk of clinically significant pocket hematoma and no thromboembolic events in patients undergoing elective CIED surgery.

### 4.3 Study limitations

This was a retrospective observational single-center study with its inherent limitations. Selection bias may play a role as DOAC are less often used in patients with renal dysfunction which is a known risk factor for pocket hematoma. Furthermore, we were unable to statistically correct for differences in baseline variables between groups due to the low number of events.

## 5 Conclusions

In patients with AF undergoing an elective CIED procedure, the risk of a clinically significant pocket hematoma and a systemic thromboembolic event is comparably low when using either continued VKA or interrupted DOAC.

## Declaration of Competing Interest

The authors declare that they have no known competing financial interests or personal relationships that could have appeared to influence the work reported in this paper.
